# Beneficial effects of low alcohol exposure, but adverse effects of high alcohol intake on glymphatic function

**DOI:** 10.1038/s41598-018-20424-y

**Published:** 2018-02-02

**Authors:** Iben Lundgaard, Wei Wang, Allison Eberhardt, Hanna Sophia Vinitsky, Benjamin Cameron Reeves, Sisi Peng, Nanhong Lou, Rashad Hussain, Maiken Nedergaard

**Affiliations:** 1 Center for Translational Neuromedicine, Department of Neurosurgery, University of Rochester, Rochester, NY 14642 USA; 2 Department of Radiology, Tongji Hospital, Tongji Medical College, Huazhong University of Science and Technology, Wuhan, 430030 China; 3 Center for Basic and Translational Neuroscience, University of Copenhagen, 2200 Copenhagen, Denmark

## Abstract

Prolonged intake of excessive amounts of ethanol is known to have adverse effects on the central nervous system (CNS). Here we investigated the effects of acute and chronic ethanol exposure and withdrawal from chronic ethanol exposure on glymphatic function, which is a brain-wide metabolite clearance system connected to the peripheral lymphatic system. Acute and chronic exposure to 1.5 g/kg (binge level) ethanol dramatically suppressed glymphatic function in awake mice. Chronic exposure to 1.5 g/kg ethanol increased GFAP expression and induced mislocation of the astrocyte-specific water channel aquaporin 4 (AQP4), but decreased the levels of several cytokines. Surprisingly, glymphatic function increased in mice treated with 0.5 g/kg (low dose) ethanol following acute exposure, as well as after one month of chronic exposure. Low doses of chronic ethanol intake were associated with a significant decrease in GFAP expression, with little change in the cytokine profile compared with the saline group. These observations suggest that ethanol has a J-shaped effect on the glymphatic system whereby low doses of ethanol increase glymphatic function. Conversely, chronic 1.5 g/kg ethanol intake induced reactive gliosis and perturbed glymphatic function, which possibly may contribute to the higher risk of dementia observed in heavy drinkers.

## Introduction

Chronic alcoholism adversely affects the brain, and known effects include cognitive impairment, neurotransmitter imbalances, and brain atrophy^1,2^. Neuroimaging studies have shown that white matter and gray matter volumes can decrease by as much as 10% in chronic alcoholics^1,3,4^. Conversely, the cerebrospinal fluid (CSF) content in the brain is increased in proportion to the reduction in brain volume^4^. Several acute effects of ethanol (hereafter referred to as alcohol), such as reduced cerebral glucose metabolism, rapidly normalize after alcohol withdrawal^5^. There is some evidence for reversibility of brain atrophy in ex-alcoholics^6^, albeit, the white matter volume is only partially recovered after 30 days of sobriety^3^. Heavy alcohol consumption for prolonged periods of time greatly increases the risk of developing dementia^7^, although there are conflicting reports regarding alcohol consumption and the risks for Alzheimer’s dementia^8^ and Parkinson’s disease^9^. These neurodegenerative disorders share certain pathologies such as intracerebral accumulation of amyloid beta (Aβ) and tau, or α-synuclein protein aggregates, respectively^10^.

While it is indisputable that excessive alcohol consumption is a health hazard, several studies have found that low doses of alcohol might be beneficial for health. Thus, comorbidity seems to follow a J-shaped curve as a function of alcohol intake, suggesting that small amounts of alcohol are indeed beneficial, but that high amounts are detrimental to health. Reduced risk of cardiovascular diseases as well as a number of cancers and increased cerebral blood flow are among the health benefits attributed to low alcohol consumption^11–14^. Although even moderate alcohol intake increases the risk of a small subset of cancers^11^, epidemiological studies have found that low alcohol consumption nonetheless reduces overall mortality from all causes^15,16^. Low and moderate intake of alcohol have also been shown to reduce the risk of dementia^17^.

We have in recent years described a highly-organized system of CSF-interstitial fluid exchange which we denominate as the glymphatic system^18–20^. This fluid transfer pathway subserves clearance of waste products and metabolites from the intercellular space of the brain, by draining into lymphatic vessels of the head and neck to transfer CSF proteins and metabolites to the general circulation for ultimate degradation in the liver^18,21,22^. Since the glymphatic system plays a key role in clearance of potentially neurotoxic proteins including Aβ^18^ and tau^23^, we here investigated glymphatic function in awake mice that had undergone acute and chronic ethanol exposure or chronic exposure followed by 24 hours of withdrawal. We report a reduction in glymphatic function resulting from acute and chronic binge-levels of alcohol exposure. Unexpectedly, glymphatic activity was increased in mice receiving a low acute or low chronic dose of alcohol.

## Results

### Acute exposure to small amounts of alcohol boosts glymphatic function

**Figure 1:**
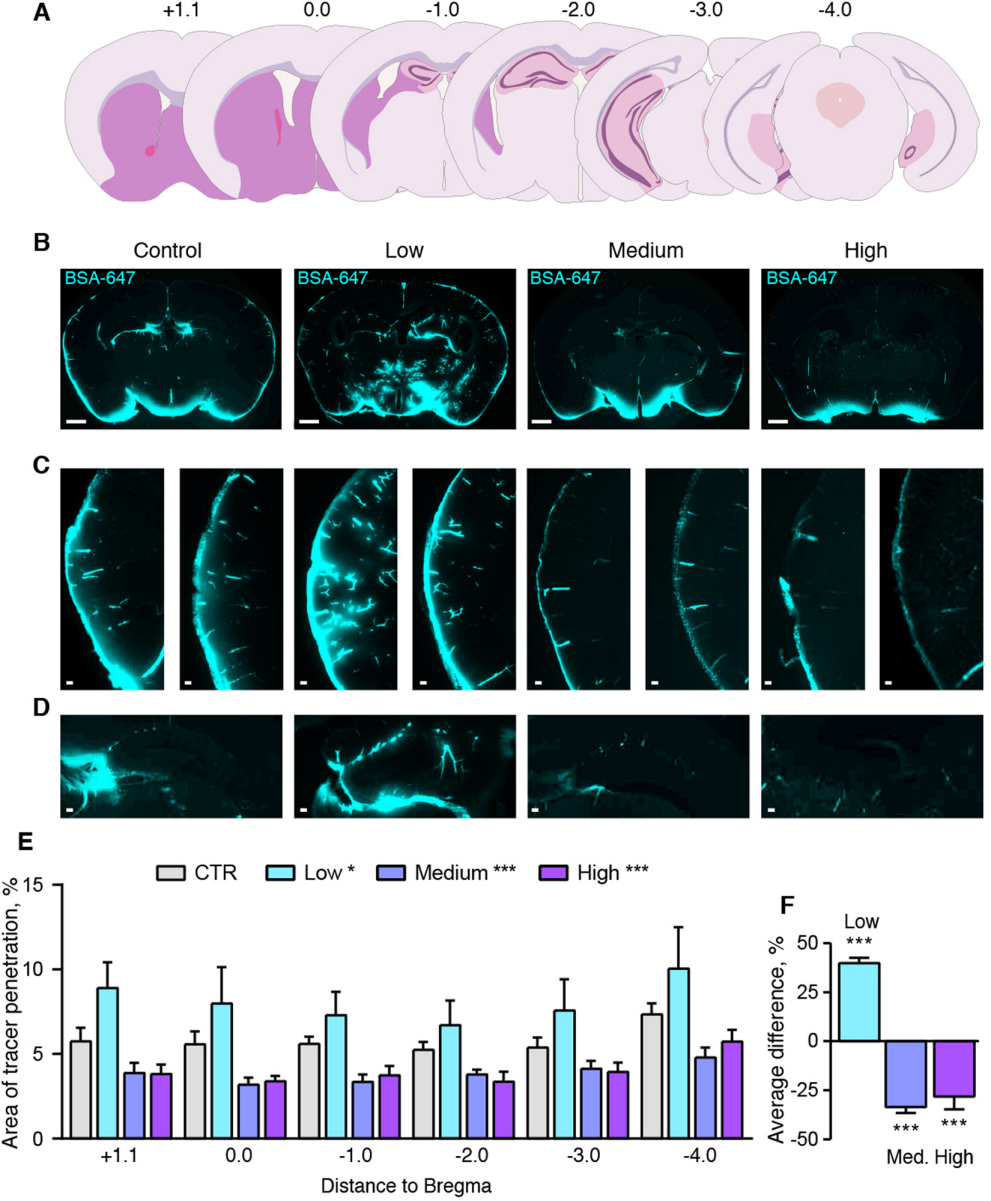
Low or high alcohol doses have opposite effects on CSF tracer penetration. (**A**) Representation of the coronal brain slices used in analysis to assess glymphatic function, with anterior-posterior distance indicated in mm in relation to bregma. (**B**) Coronal sections of mouse brain at 30 minutes after cisterna magna injections of Alexa-647-conjugated bovine serum albumin (BSA-647) in awake mice. Scale bars: 1 mm. (**C**) Tracer influx in the cortex in two different representative mice. Scale bars: 100 µm. (**D**) Influx in the hippocampus at bregma −1 mm. Scale bars: 100 µm. (**E**) Area covered by tracer influx in coronal brain slices collected 30 minutes after cisterna magna tracer injection in mice given saline or alcohol 15 minutes before they were injected with CSF tracer. X-axis indicates the distance to bregma. CTR, saline control; low, medium, high, 0.5, 1.5 and 4 g/kg ethanol, respectively. 2-way ANOVA compared to control. (**F**) Average difference compared to control for all brain slices analyzed. One-way ANOVA compared to control. *p < 0.05, **p < 0.01, ***p < 0.001. Bar graphs represent mean and standard error of the mean (SEM) of 7–9 mice per group.

Awake, behaving C57Bl6 mice received low, intermediate, and high doses of alcohol (0.5, 1.5 and 4 g/kg [intraperitoneally, I.P.], respectively) 15 min before injection of CSF tracers into the cisterna magna compartment via a cannula implanted 24 hrs earlier. The brains were quickly harvested at 30 min after the tracer injection and immersion-fixed followed by preparation of coronal vibratome sections. Microscopic examination revealed robust tracer influx along paravascular pathways surrounding predominantly cortical arterioles and the anterior choroidal artery in all coronal slices analyzed (+1.1, 0, −1, −2, −3, −4 mm from bregma) (Fig. 1A,B). Tracer influx was also noted in and around the hippocampal formation (Fig. 1C). On average, the low-dose alcohol group (0.5 g/kg) exhibited a 39.8 ± 2.8% increase in CSF tracer influx compared to vehicle controls (2-way ANOVA p < 0.05 for all coronal series, one-way ANOVA for average, p < 0.001) (Fig. 1E,F). In contrast, intermediate (1.5 g/kg) and high acute alcohol doses (4 g/kg) suppressed glymphatic function by a mean of 33.5 ± 3.0% and 28.0 ± 6.0%, respectively (both p < 0.001 for all series and average) (Fig. 1E,F). Interestingly, the suppression of glymphatic influx in the intermediate and high dose of alcohol were most pronounced along the cortical vessels, with lesser reduction in tracer influx via thalamic or hypothalamic influx pathways (Fig. 1B–D). The suppressive effects of alcohol on cortical CSF tracer influx were present across the rostral to caudal axis of the brain. Taken together, these observations show that a low acute dose of alcohol improves glymphatic function, whereas higher acute doses of alcohol have a suppressive effect on glymphatic function assessed with the CSF tracer. The acute glymphatic suppression from moderate and high dose alcohol may be due simply to lowered cardiac output from alcohol intoxication^24^ resulting in reduced pulse pressure, which has previously been identified as a driver of CSF flux along the periarterial channels^25^.

### Chronic low dose alcohol intake increases glymphatic function while chronic medium dose is inhibitory

**Figure 2:**
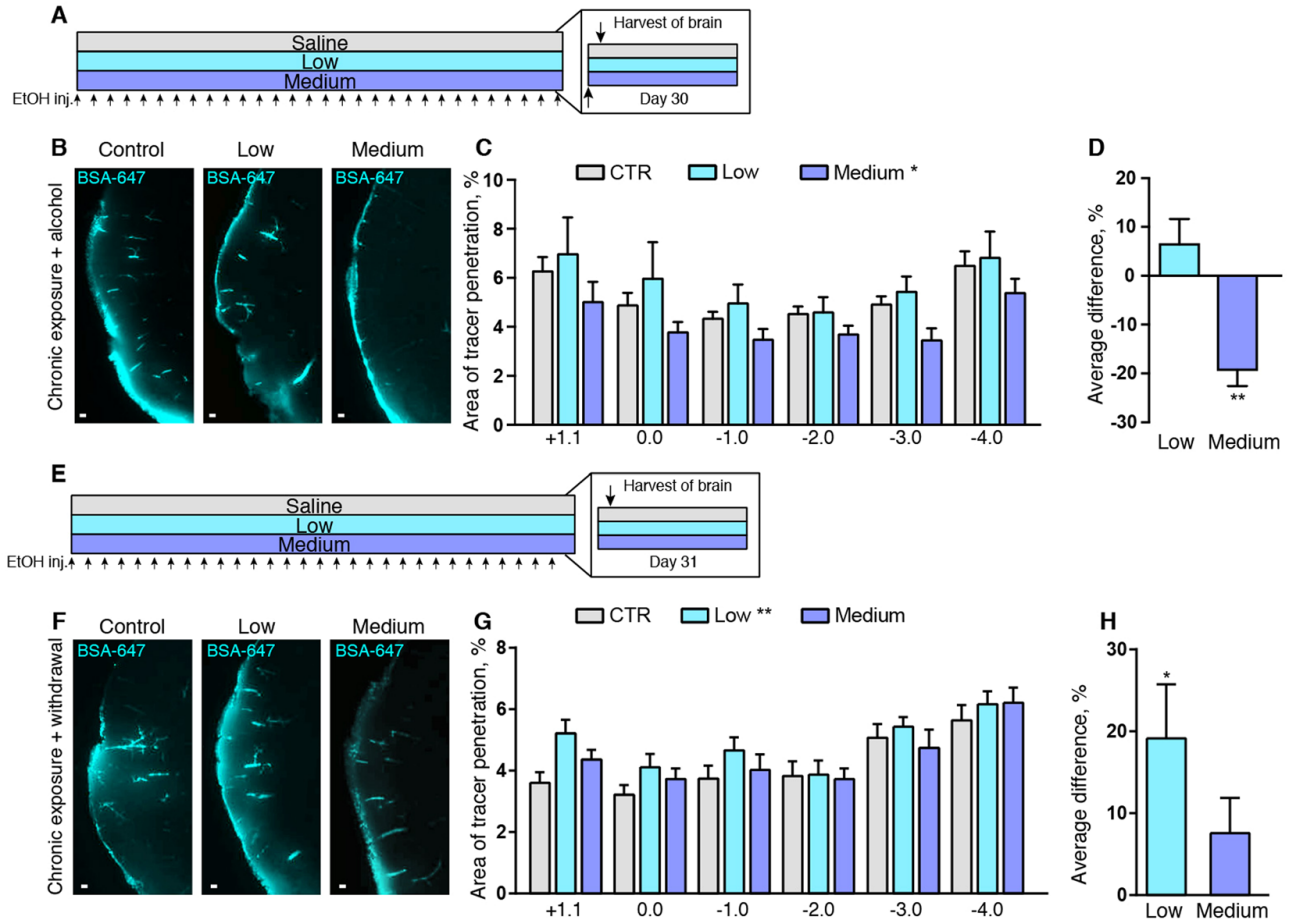
Withdrawal from long term alcohol exposure increases glymphatic function. (**A**) Timeline diagram of experiment. Arrows on the bottom indicate injections of alcohol (EtOH) or saline. This experiment was performed 15 minutes after the last of 30 days of daily injections. (**B**) Tracer influx in the cortex 30 minutes after cisterna magna injections of Alexa-647-conjugated bovine serum albumin (BSA-647) in awake mice with chronic alcohol exposure. Scale bars: 100 μm. (**C**) Area covered by tracer influx in coronal brain slices 30 minutes after cisterna magna injection. X-axis indicates the distance to bregma. 2-way ANOVA compared to control. (**D**) Average difference compared to control condition for all coronal brain slices analyzed. One-way ANOVA compared to control. (**E**) Timeline diagram of experiment. Arrows on the bottom indicate alcohol (EtOH) or saline injections. This experiment was performed 24 hours after the last of 30 daily tracer injections. (**F**) Tracer influx in the cortex 30 minutes after cisterna magna injections of Alexa-647-conjugated bovine serum albumin (BSA-647) in awake mice with 24 hours withdrawal from chronic alcohol treatment. Scale bars: 100 μm. (**G**) Quantifications of tracer influx in mice treated with alcohol or saline for 30 days, measured 24 hours after the last treatment. 2-way ANOVA compared to control. (**H**) Average difference compared to control for all coronal brain slices analyzed. One-way ANOVA compared to control. CTR, saline; low, 0.5 g/kg ethanol; medium, 1.5 g/kg ethanol. *p < 0.05, **p < 0.01, ***p < 0.001. Bar graphs represent mean and standard error of the mean (SEM) of 7–11 mice per group.

To assess the effects of chronic alcohol consumption, we next treated mice with low and intermediate doses of alcohol for 30 days and assayed glymphatic function in the awake state shortly after the last alcohol administration (Fig. 2A). The group of mice that received the low chronic dose (0.5 g/kg) exhibited a non-significant trend towards increased CSF tracer influx in coronal slices along the caudal-rostral axis compared with vehicle-treated controls (Fig. 2B,C). When averaging all the slice series, there was a non-significant 6.4 ± 5.2% influx increase in the low dose alcohol group (Fig. 2D). Conversely, prolonged administration of the medium dose (1.5 g/kg) significantly suppressed mean glymphatic function in caudal-rostral slices by 19.2 ± 3.3% (one-way ANOVA, p < 0.001) (Fig. 2C,D). We did not include the high dose in our long-term exposure experiments, as this dose may introduce confounding factors such as hepatic inflammation and steatosis^26,27^, and in our pilot study, chronic exposure to the high dose of alcohol had a mortality rate of 40%.

To assess whether the effects of chronic alcohol on glymphatic function are reversed after withdrawal, we administered alcohol daily for 30 days and then performed glymphatic function assays at 24 hours after the last dose of alcohol (Fig. 2E). Interestingly, the 24-hour withdrawal from low dose chronic alcohol treatment increased glymphatic function by an average of 19.1 ± 6.6% compared to saline (one-way ANOVA, p < 0.05) (Fig. 2F–H). Withdrawal from the intermediate dosage of alcohol for 24 hours was met with a restoration of glymphatic function to normal levels based on two comparisons. We first considered coronal levels at 1 mm intervals extending from −4 to +1.1 mm relative to bregma in the anterior-posterior direction (two-way ANOVA), and next calculated the mean uptake in all slices (one-way ANOVA). Neither of these comparisons revealed any difference in glymphatic function between saline and medium dose alcohol groups at 24 hours after the last of 30 daily alcohol administrations (Fig. 2G,H).

**Figure 3:**
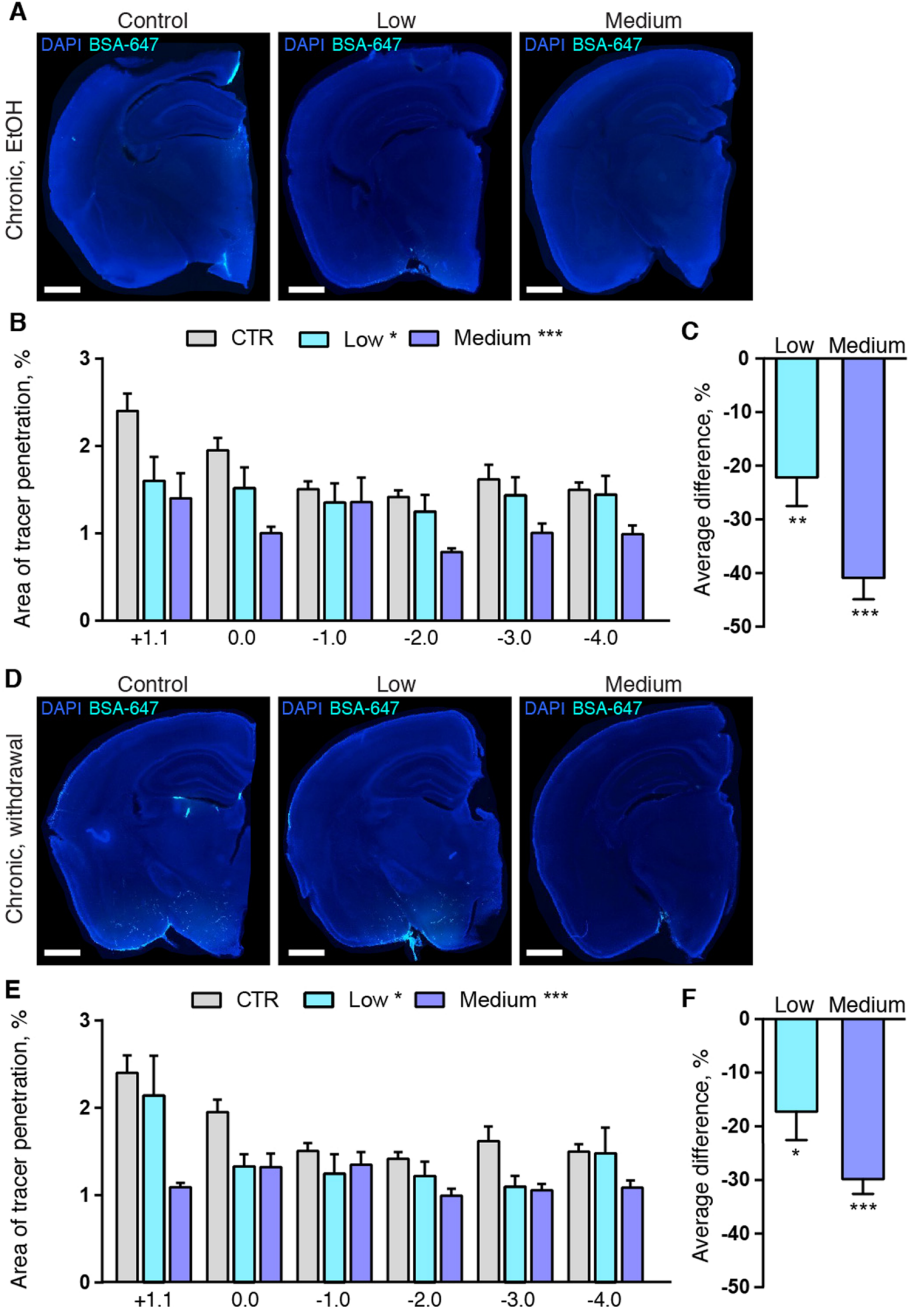
Tracer clearance is improved in alcohol-treated mice. (**A**) CSF tracer in coronal slices of left brain hemisphere at 180 min after injection in mice chronically treated with saline (CTR), low (0.5 g/kg) and medium (1.5 g/kg) doses of alcohol with the last dose of alcohol given immediately before tracer injection (EtOH), (**B**) quantification of tracer in slices at the indicated distances to bregma (2-way ANOVA) and (**C**) average difference compared to control condition for all coronal brain slices analyzed (one-way ANOVA). (**D**) CSF tracer in coronal slices of left brain hemisphere at 180 min after injection in mice chronically treated with saline (CTR), low and medium dose of alcohol with the last dose of alcohol given 24 hours before tracer injection (withdrawal), (**E**) quantification of tracer in slices at the indicated distances to bregma (2-way ANOVA) and (**F**) average difference compared to control condition for all coronal brain slices analyzed (one-way ANOVA). *p < 0.05, **p < 0.01, ***p < 0.001. Bar graphs represent mean and standard error of the mean (SEM) of 5 saline mice, 10 low and 10 medium alcohol dose mice per group for both EtOH and withdrawal experiments.

To test whether tracer washout was affected by chronic low and medium alcohol intake, we next quantified tracer accumulation following three hours of circulation^28^. Since influx of CSF tracers peaks after approximately 30–60 min, the prolonged tracer circulation time can be used to study clearance without invasive intraparenchymal injections^28^. The CSF tracer signal after three hours was reduced by 22.2 ± 5.3% (p < 0.001) in the low dose and by 40.9 ± 4.0% (p < 0.001) in the medium chronic alcohol dose mice while affected by alcohol (Fig. 3A–C, Supplemental Fig. 1A). At 24-hour withdrawal from chronic treatment, the percent of brain area containing fluorescence signal was on average 17.2 ± 5.3% and 29.9 ± 2.7% lower with low and medium dose of alcohol compared with saline, respectively (p < 0.05 and p < 0.001, respectively) (Fig. 3D–F, Supplemental Fig. 1B). These observations were consistent with increased glymphatic clearance in the low dose alcohol group, since tracer influx at 30 min was higher than the saline group (Fig. 2B), yet the tracer amount was reduced to lower levels at three hours compared with the saline control mice (Fig. 3A–F, Supplemental Fig. 1A,B). Since tracer influx was reduced at 30 min in the chronic moderate alcohol group, the reduced tracer accumulation after three hours accumulation cannot be unambiguously attributed to increased clearance.

Clearance of tracer from the brain could also be confounded by leakage via the blood-brain barrier (BBB). There are conflicting reports on the effect of chronic alcohol exposure on BBB function. Some reports in laboratory animals have found an increase in BBB permeability due to alcohol intake but only when another challenge, such as food withdrawal or lipopolysaccharide (LPS) was added^29,30^. Efflux of CSF tracer usually happens by drainage via the peripheral lymphatic system^31^. However, a defect in the BBB could potentially cause leakage of tracer directly into the blood circulation. To test whether increased dye shunting to blood occurred in the alcohol groups, we evaluated the signal derived from the CM-injected tracer in the livers of mice receiving saline or alcohol treatment (Supplemental Fig. 2A,B). The fluorescence signal was increased compared to the background signal in non-tracer injected mice (Supplemental Fig. 2C), but there was no difference in the dye intensity between the tracer-injected groups, neither for chronic alcohol administration, nor upon withdrawal after chronic administration (p = 0.23 and p = 0.74, respectively) (Supplemental Fig. 2D).

### The effect of alcohol on glymphatic function is not mediated by changes in sleep

**Figure 4:**
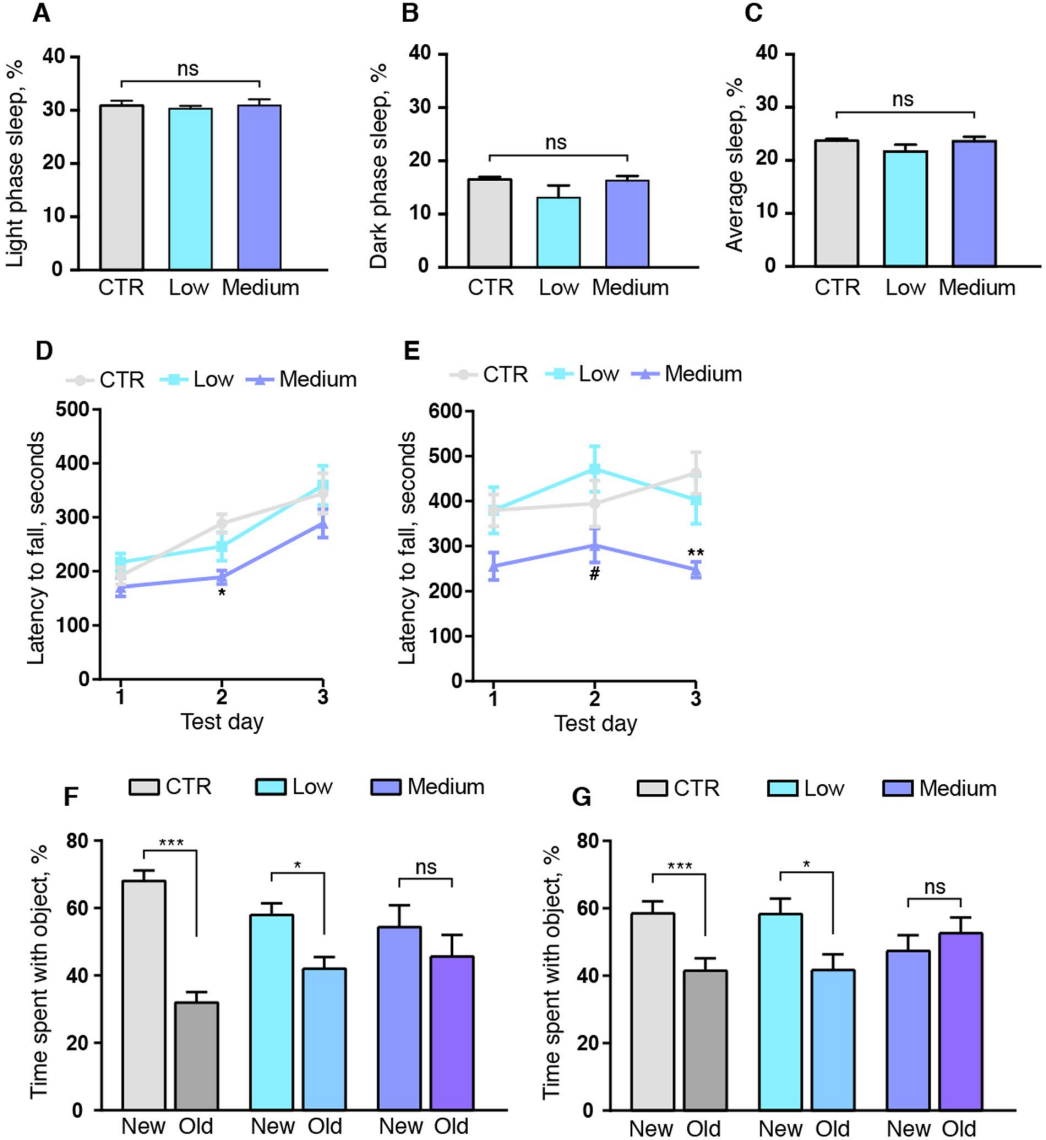
Long term exposure to 1.5 g/kg alcohol does not affect sleep but impairs motor and learning skills. Immobility-defined sleep duration in saline (CTR), 0.5 g/kg (low) and 1.5 g/kg (medium) alcohol treatment groups did not show any difference between the groups for (**A**) light phase sleep, (**B**) dark phase sleep or (**C**) mean sleep duration. Alcohol was administered at 9 am, three hours into the light phase. Latency to fall of the Rota-Rod (**D**) at 24 hours after withdrawal from 30 days chronic alcohol exposure or (**E**) with acute alcohol challenge following 30 days of chronic exposure. 2-way ANOVA with Tukey test compared to saline treatment or low dose alcohol. *p < 0.05, **p < 0.01 compared to saline. #p < 0.05 compared to low dose. Novel object test at (**F**) 24 hours after withdrawal from chronic alcohol exposure and (**G**) acutely affected by alcohol following 15–28 days of chronic exposure. New objects had never previously been seen by the mouse, and old objects had been introduced to the mouse for first examination for ten minutes at a time one hour prior to testing on the same experimental day. Student’s t-test comparing percentage time spent exploring new and old object. CTR, saline; low, 0.5 g/kg ethanol; medium, 1.5 g/kg ethanol. Ns, not significant, *p < 0.05, **p < 0.01, ***p < 0.001. Bar graphs represent mean and standard error of the mean (SEM) of 4 mice per group for sleep analysis, 11–21 mice per group for novel object test and plots represent mean and SEM of 15–25 mice for Rota-Rod test.

Since glymphatic function is highly regulated by the sleep cycle^20,32^ we investigated whether the beneficial effects of low dose alcohol on the glymphatic system in mice was due to changes in average sleep times. We used an immobility-defined sleep assay to non-invasively measure the fraction of sleep versus waking in the saline, low and medium alcohol mouse groups^33,34^. This assay correlates with 94% accuracy to sleep measured by standard EEG/EMG procedures^33–35^. Following three days of acclimatization to their new home cage, we analyzed sleep during a 24 hour period. The low and medium dose alcohol group did not show any difference in light phase, dark phase or average sleep time compared to the saline group (1-way ANOVA, p = 0.86, p = 0.23 and p = 0.26, respectively) (Fig. 4A–C). Neither could we detect any group differences for the mean number or length of sleep bouts for low or medium alcohol (p = 0.13 and p = 0.89, respectively, not shown). We note that the mice were closely monitored throughout the glymphatic experiments, which span 30 minutes (for influx) and 180 minutes (for clearance), and were not allowed to sleep during the period spanning from tracer injection to brain harvest.

### 24 hours of withdrawal from chronic binge-level exposure alcohol does not entirely restore motor function

Comparisons of preclinical studies of alcohol toxicity are confounded by factors influencing ethanol elimination, notably including food intake, which was not controlled in this study^36^. However, we did investigate the effects of alcohol on motor performance of the mice, using a Rota-Rod with a rotation rate increasing from 5 to 40 rpm^37^. Results of two trials were averaged and repeated three times on separate days. We first performed the withdrawal trials in the mice with chronic alcohol treatment followed by 24 hours withdrawal; the mean Rota-Rod performance in saline-treated mice was then 165.9 ± 13.0 seconds (Fig. 4D). The saline and low alcohol groups showed improvement in the second trial, the saline being able to remain on the rotating rod for 246.3 ± 13.3 seconds on day 2, but motor performance did not improve after withdrawal in the medium dose group. The latency to fall off the Rota-Rod upon withdrawal was 67.2 ± 15.3 seconds briefer in the medium dose alcohol group than in the saline-treated group (p < 0.05). Overall, the medium-dose group showed a 27.3 ± 6.2% decrease in time to failure in comparison to the saline group. All groups showed an improvement in motor performance between days two and three, and there were no significant group differences on day three. Performance by the low-dose and control groups did not differ in any trial.

We then assessed mice on the Rota-Rod after chronic alcohol exposure with final alcohol administration at 15 min before the trial. The saline-treated group stayed on the rod for 384.8 ± 29.2 seconds at day 1, 345.2 ± 30.3 seconds at day 2, and 406.9 ± 34.5 seconds at day 3. While there was no difference between the saline and the low dose group in any of the trials, performance differed between the low and the medium dose group on day 2, when the medium group fell off the rod 128.8 ± 38.3 seconds sooner (p < 0.05), corresponding to a 37.3 ± 11.2% decrease in Rota-Rod endurance. On day 3, the mice that had received the medium dose of alcohol fell off the rod 147.1 ± 17.6 seconds before the saline-treated mice (p < 0.01) (Fig. 4E), corresponding to a 31.7 ± 7.6% decrease in comparison to the saline group. Thus, the low dose of alcohol did not affect motor skills after acute alcohol challenge, while the medium dose perturbed motor skill with acute alcohol challenge and upon 24 hours of withdrawal.

### Chronic administration of alcohol impairs learning and memory both under the influence and during withdrawal

After chronic alcohol administration, the mice also underwent a novel-object test for learning and memory. Each group of mice was randomized into three sub-groups and left to explore two objects in an open field. In the test phase, the mice were presented with one familiar and one novel object, and their interactions recorded.

24 hours after the last alcohol or saline administration, the saline group spent a significantly longer time exploring the new object compared to the familiar object (p < 0.001), similar to results in the low dose alcohol group (p < 0.01) (Fig. 4F). In contrast, the medium alcohol dose group did not spend significantly more time exploring the novel object in the withdrawal phase (p = 0.35). In the experiments with acute alcohol treatment after chronic exposure, the mice in the saline and low alcohol dose groups still spent more time exploring the new object than the old object (p < 0.01, and p < 0.05, respectively) (Fig. 4G). However, the medium dose group did not show any increased interest in the novel object, suggesting that these mice suffer from learning impairment both under the influence and during alcohol withdrawal (p = 0.43).

### Alcohol exposure produces different changes in GFAP and AQP4, depending on the dose

**Figure 5:**
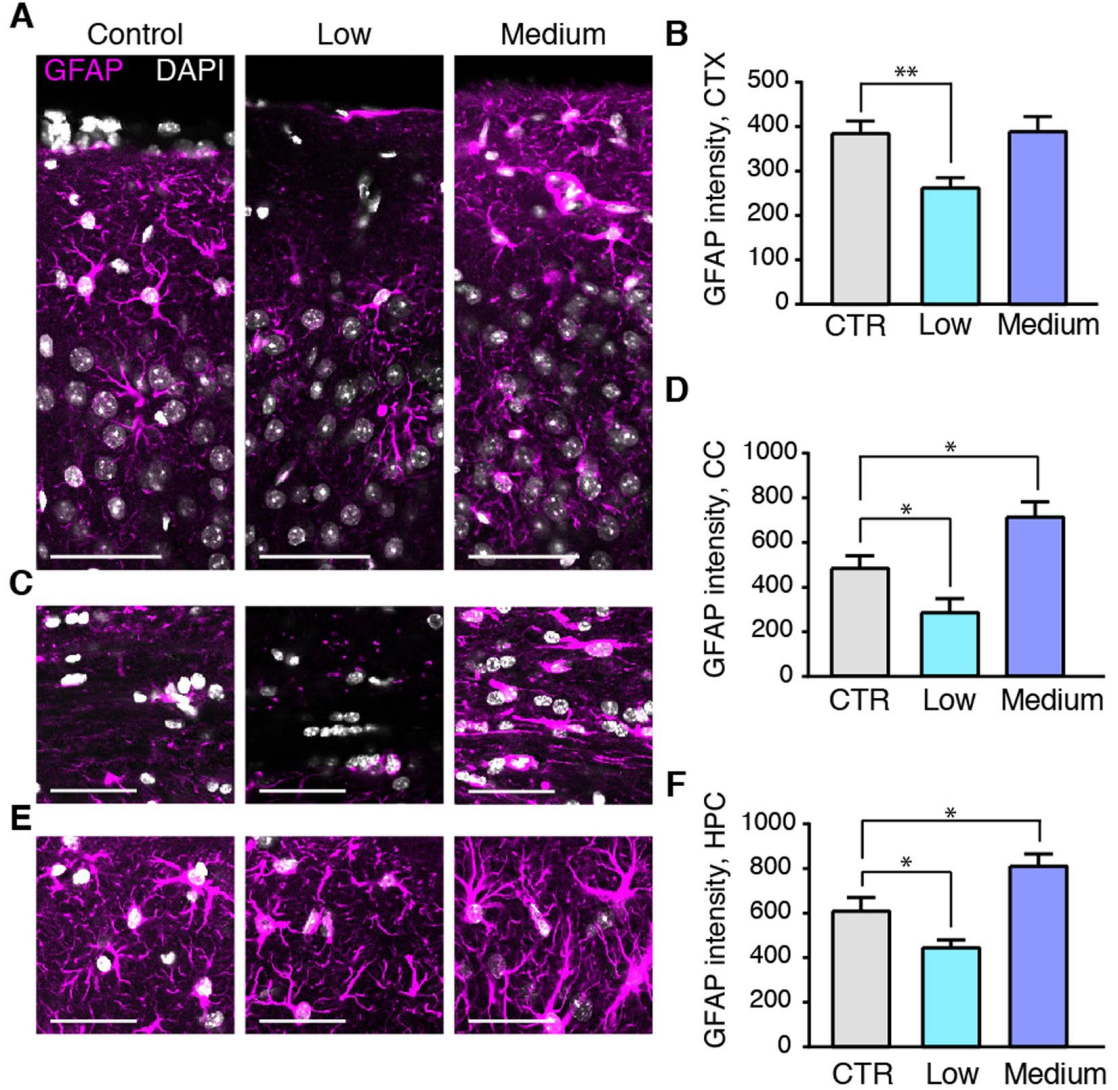
Chronic administration of 0.5 g/kg alcohol decreases GFAP, while 1.5 g/kg alcohol increases astrogliosis. Immunostaining for GFAP (astrocyte marker, magenta) and DAPI (white) and quantifications thereof in control mice and in mice chronically treated with alcohol in cortex (**A**,**B**), corpus callosum (**C**,**D**) and hippocampus (**E**,**F**). CTR, saline; low, 0.5 g/kg ethanol; medium, 1.5 g/kg ethanol. *p < 0.05, **p < 0.01, one-way ANOVA with Tukey test. Scale bars, 100 µm. Bar graphs represent mean and standard error of the mean (SEM) of 3–4 mice per group.

A range of conditions in which astrocytes acquire a reactive phenotype, characterized by hypertrophy of GFAP-positive processes, have in several prior publications been associated with reduced glymphatic function^38–40^. We evaluated GFAP immunostainings of mice chronically treated with low or medium alcohol doses in comparison to control groups. Chronic exposure to the medium dose of alcohol did not change astrocytic GFAP-positive processes in the cortex (Fig. 5A,B). However, chronic exposure to the medium alcohol dose upregulated GFAP immunostaining-intensities in the corpus callosum (Fig. 5C,D) and hippocampus (Fig. 5E,F) (p < 0.05 and p < 0.05, respectively). Interestingly, treatment with the low dose of alcohol significantly *decreased* GFAP immuno-staining in cortex, corpus callosum, and hippocampus (p < 0.01, p < 0.05, p < 0.05, respectively) (Fig. 5A–F).

**Figure 6:**
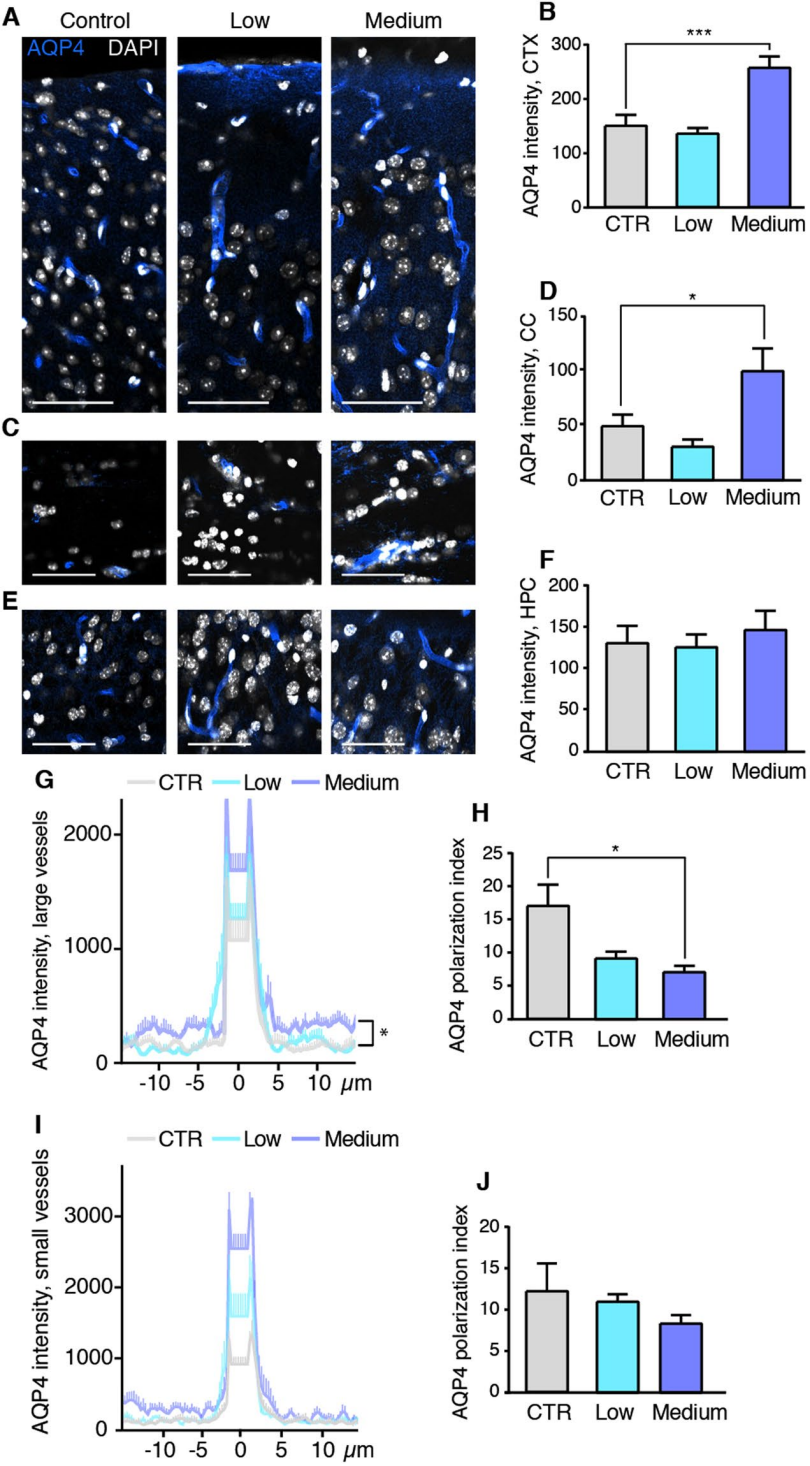
Chronic administration of medium dose alcohol increases AQP4 expression and reduces AQP4 polarity. Immunostaining for AQP4 (blue) and DAPI (white) and quantifications thereof in control mice and mice with chronic alcohol treatment in cortex (**A**,**B**), corpus callosum (**C**,**D**) and hippocampus (**E**,**F**). Line-plots of AQP4 immuno-intensity across arterioles (**G**) in the cortex, and calculation of the peak-to-baseline level (AQP4 polarization index) (**H**). Line-plots of AQP4 immuno-intensity across capillaries (**I**) in the neocortex and calculation of the AQP4 polarization index (**J**). CTR, saline; low, 0.5 g/kg ethanol; medium, 1.5 g/kg ethanol. *p < 0.05, **p < 0.01, ***p < 0.001, one-way ANOVA with Tukey test. Scale Bars, 100 µm. Bar graphs and plots represent mean and standard error of the mean (SEM) of 3–4 mice per group.

We next investigated the astrocyte-specific water channel AQP4, which is located specifically in the plasma membrane of astrocyte end-feet^41–43^. AQP4 has previously been shown to play a crucial role in glymphatic function^18,20,38,44^. Chronic daily exposure to medium alcohol doses increased the general level of parenchymal AQP4 in astrocytes of the cortex and corpus callosum (p < 0.001 and p < 0.05, respectively) (Fig. 6A–D), but AQP4 expression was unaffected in the hippocampus (Fig. 6E,F). There were no corresponding effects of low dose of alcohol relative to the saline group.

An analysis of the AQP4 polarization index^38^ showed that the polarization (peak intensity value at the end-feet divided by the tissue baseline intensity) was decreased in the medium alcohol group (p < 0.05), while there was no significant change in the low alcohol group (Fig. 6G,H). The reduction in polarity in the medium alcohol group was attributable to an increase in the baseline of AQP4 expression, i.e. the parenchymal expression (p < 0.001) (Fig. 6G). AQP4 polarization around capillaries did not differ in between the groups (Fig. 6I,J).

Not unexpectedly, acute alcohol exposure did not change the level of GFAP or AQP4 compared to the saline control group for neither cortex, corpus callosum or hippocampus (p = 0.99, p = 0.52, and p = 0.77 for GFAP and p = 0.44, p = 0.27, and p = 0.36 for AQP4, respectively, data not shown) suggesting that the effect of acute alcohol exposure on glymphatic function is not mediated by a change in GFAP or AQP4 expression. However, the inhibitory effects of chronic medium alcohol exposure on glymphatic function may be in part mediated by the increased reactive gliosis and mislocation of AQP4 water channels in astrocytes^38,39^. Conversely, the beneficial effects of low doses of alcohol on glymphatic activity might point to a novel mechanism whereby decreased GFAP expression facilitates glymphatic system function. On the other hand, it is also possible that the decrease in GFAP is not causally linked to the increase in glymphatic activity.

### Low doses of alcohol exposure change the cerebral cytokine profile

**Figure 7:**
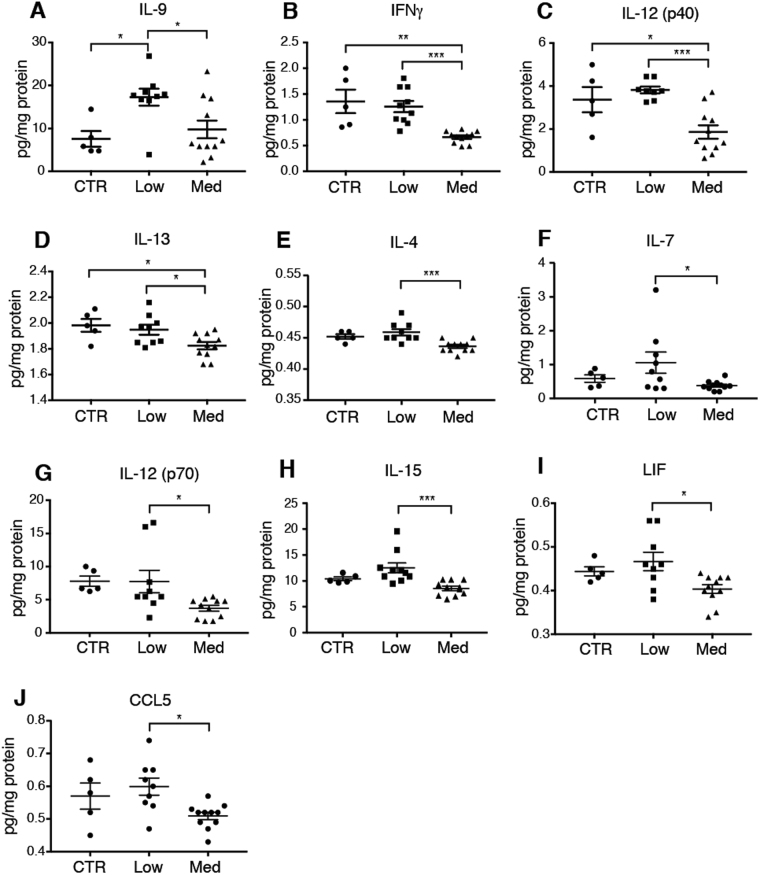
Alcohol changes cytokine levels, depending on the dose. (**A**–**J**) Cytokine levels measured at the protein level in perfusion-desanguinated brain of mice chronically treated with alcohol, with the last dose of alcohol given on the same day as the experiment. CTR, saline; low, 0.5 g/kg ethanol; medium, 1.5 g/kg ethanol. IL, interleukin; IFNγ, interferon gamma; LIF, leukemia inhibitory factor; CCL 5, Chemokine C-C motif ligand 5. *p < 0.05, **p < 0.01, ***p < 0.001, one-way ANOVA with Tukey test. Mean and standard error of the mean (SEM) is indicated on the plots of individual values. 5, 10 and 11 mice per group for saline, low and medium alcohol groups, respectively. Some readings were out of range (not within the standard curve) and could therefore not be reported here.

Gliosis often goes hand in hand with neuroinflammation^45^. The up- and downregulation of GFAP in astrocytes exposed to prolonged medium and low levels of alcohol, respectively, led us to ask whether alcohol affects the inflammatory status of the brain. Depending on the dose, alcohol has different effects on systemic inflammation with a tendency to a decrease in peripheral inflammation with light intake and conversely an increase in inflammation in chronic high intake^46,47^. However, there are no reports describing the effect of prolonged exposure to low doses of alcohol on the CNS cytokine profile. We therefore treated groups of mice with low and medium doses of alcohol for four weeks and collected the brains after a brief transcardial perfusion with PBS to avoid contaminating the brain samples with systemic cytokines. Interestingly, the blinded cytokine analysis of the brain showed that interleukin (IL)-9 was elevated to 228.2 ± 26.1% of saline values (p < 0.05) in the low dose alcohol group, but unchanged in the medium dose alcohol group (p = 0.78) (Fig. 7A). IL-9 is a pleiotropic cytokine expressed by thymocyte helper 2 (Th2) or T regulatory (Treg) cells; its expression can be induced by the anti-inflammatory cytokines TGFb and IL-4, but is typically reduced by classic pro-inflammatory cytokines such as interferon gamma (IFNγ) and IL-23^48^. Since IL-9 is produced by and acts on T cells and since no cell type native to the brain express IL-9 receptors, it is not likely to affect the brain directly^49,50^. The analysis of the medium alcohol dose group depicted a general pattern of down-regulation of pro-inflammatory cytokines IFNγ (−49.1 ± 2.6%; p < 0.01), and IL-12-p40 (−55.3 ± 9.3%; p < 0.05) as well as the anti-inflammatory cytokine IL-13(−92.1 ± 1.4%; p < 0.05) compared with saline treated mice (Fig. 7B–D). IL-12 acts upstream of the IFNγ pathway by inducing IFNγ secretion from natural killer (NK) cells and T cells^51^. IL-13, which showed a modest but significant decrease of 7.9 ± 1.4% (p < 0.05) in the medium alcohol group dose, is expressed by microglia^52^. The average level of many cytokines was lower in the medium dose alcohol group compared to the low dose alcohol group: IFNγ, IL-4, IL-7, IL-9, IL-12 p40, IL-12 p70, IL-13, IL15, leukemia inhibitory factor (LIF), and CCL5 (Fig. 7E–M). These cytokines are not classical astrocyte cytokines^45^, suggesting that chronic alcohol exposure does not affect astrocytes’ inflammatory state directly^53^, but decreases the general level of cytokines in the brain. We did not detect any differences between the groups in the following cytokines or growth factors: eotaxin, G-CSF, GM-CSF, IL-1a, IL-1b, IL-2, IL-3, IL-5, IL-10, IL-17, LIX, MCP-1, M-CSF, MIG, MIP-1a, MIP-1b, MIP-2, VEGF, IL-11, TGFb, MCP-1 (not shown). Thus, we find that the low alcohol dose did not induce significant changes in the cytokine profile except for IL-9, while chronic exposure to 1.5 g/kg alcohol per day was linked to a distinct cytokine pattern, with prominent downregulation of cytokines.

## Discussion

We here investigated the effect of acute and chronic alcohol treatments on glymphatic function in mice, finding that acute alcohol intake potently alters glymphatic function in the awake state depending on the dosage. Intermediate alcohol exposure (1.5 g/kg), corresponding to 7.9 standard daily drinks daily (NIH definition; 12-ounce beers containing 5% alcohol, or 5-ounce glasses of wine containing 12% alcohol for a person weighing 70 kg), decreased glymphatic function following both acute and 30 days of chronic exposure. The suppression of glymphatic function was however not permanent, because glymphatic function was restored at 24 hours after termination of chronic moderate alcohol administration. A very high dose of alcohol (4 g/kg), corresponding to 21 standard drinks per day, also acutely reduced glymphatic function. Unexpectedly, however, the low dose of alcohol (0.5 g/kg) significantly improved glymphatic activity, acutely and after 30 days of chronic exposure. The combination of both increased CSF tracer influx and more robust reduction of CSF tracer during prolonged circulation of the CSF tracer suggest that glymphatic clearance capability is increased by low doses of alcohol.

The data presented here on effects of alcohol on the glymphatic system seemingly matches the J-shaped model relating dose effects of alcohol on general health and mortality, whereby low doses of alcohol are beneficial, while excessive consumption is detrimental to overall health. Low-to-moderate alcohol intake is associated with a lesser risk of dementia^17^, while heavy drinking for many years confers an increased risk of cognitive decline^54^. Daily intake of alcohol for 30 years at doses scalable to those in the present study reduces human hippocampal volume by 3.4–5.8% compared to abstainers^55^. Naturally, this study performed in mice should not be viewed as a recommendation for alcohol consumption guidelines in humans. However, our analysis is the first report of both increased glymphatic function combined with lowered expression of GFAP resulting from light alcohol intake. This observation may present a novel cellular and physiological mechanism contributing to the delay in onset of dementia in subjects with light alcohol intake, namely through enhanced glymphatic clearance. In this exploratory study, we did not establish why GFAP expression was significantly lower in the low dose alcohol group, but we do note that the mice in this study did not have access to a running wheel in their otherwise enriched housing environment. Sedentary lifestyle is associated with low grade inflammation^56,57^, and exercise can decrease GFAP expression in aged mice^40^. It is thus possible that the low dose of alcohol reduced neuroinflammation. However, low doses of alcohol also increase arterial pulsatility^58^, which is known to facilitate glymphatic influx^25^. Thus, multiple factors may contribute to the beneficial effect of the low alcohol dose on glymphatic function.

**Figure 8:**
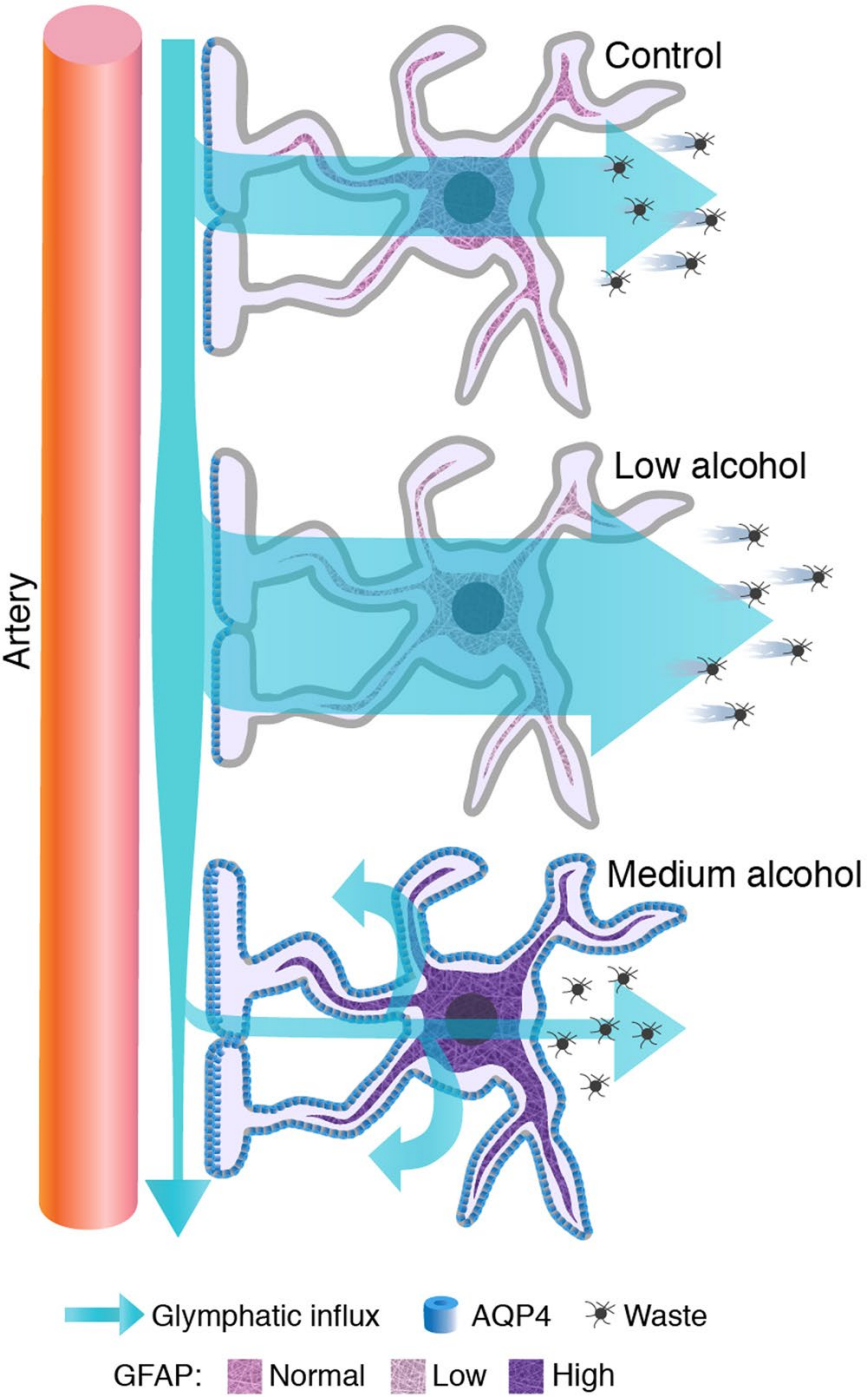
Schematic representation of the results. Astrocytes in normal physiological conditions facilitate the influx of CSF to perivascular pathways. Chronic exposure to 0.5 g/kg (low dose) of alcohol per day downregulates GFAP expression and leads to increased glymphatic function. Chronic exposure to 1.5 g/kg alcohol (binge drinking level) increases AQP4 expression in the non-end-foot processes, increases GFAP expression, and impairs glymphatic function.

The effects of alcohol on the brain did not exhibit a simple case of dose-dependence. Chronic exposure to binge-level doses of alcohol induced a downregulation of several cytokines, including IL-12, which is upstream of IFNγ in T cells, and is also expressed by microglia. Astrocytes are potent regulators of microglial IL-12p40 expression^59^, suggesting several possible mechanisms by which alcohol might affect IL-12, i.e. either by acting directly on microglia or indirectly through astrocytes. Blood contains orders of magnitude higher concentrations of cytokines than the brain and, to our knowledge, this is the first study that has analyzed cerebral cytokines following transcardial perfusion with PBS to remove blood. Thus, it is difficult to compare our present observations with prior literature. Nevertheless, the analysis presented here depicts a broad suppression of cytokines in brain similar to that in prior reports on high chronic alcohol exposure suppresses immunity^60,61^, while the effect of the low dose alcohol was minimal. Interestingly, the effects of alcohol on inflammation may be context-dependent. The general suppression of peripheral cytokines by alcohol can rapidly change to a more pro-inflammatory cytokine profile when the immune system is challenged^62^. It is important to note that the moderate dose of alcohol increased GFAP expression, lowered the polarity of AQP4 expression on astrocytes facing large arterioles as previously reported^63,64^, concomitant with a significant suppression of glymphatic function (Fig. 8). Widespread astrogliosis and abnormal subcellular localization of AQP4 water channels are associated with perturbed glymphatic clearance^38^, as revealed by tracer studies in mice. In CNS, AQP4 is expressed exclusively in astrocytes, with a highly polarized distribution in the vascular end-feet^41–43^. Polarity of AQP4 toward vascular end-feet has a functional significance in relation to CSF inflow and clearance of Aβ^18^. APP/PS1 mice with AQP4 KO have increased accumulation of Aβ compared to mice with AQP4^65^. It is therefore conceivable that alcohol-induced changes in AQP4 localization may contribute to impaired amyloid clearance. Of note, several magnetic resonance imaging (MRI) studies suggest that a functional glymphatic system also exists in the human brain^66–68^. Due to the invasive nature of assessing glymphatic activity in humans, we rely on AQP4 polarization as a proxy for glymphatic function. Supporting data for the role of AQP4 in human disease is that Alzheimer’s disease patients have decreased perivascular to parenchymal ratio of AQP4 expression and a linear correlation between lack of AQP4 polarization and cortical density of amyloid plaques^69^.

An additional potential explanation for the salutogenic effects of a low daily dose of alcohol on brain function may involve altered neurotransmission. The glymphatic system is highly active in the sleep state and much less active in awake animals^32^. Ethanol is a sedative that suppresses activity of ionotropic glutamate receptors^70^, which are common targets of general anesthetics^71^. Indeed, alcohol is itself a general anesthetic when given at a high dose. Thus, to minimize drug interaction between the alcohol and anesthesia, we analyzed glymphatic function in the awake state. The chief neuromodulator of glymphatic function is norepinephrine, mainly derived from the ascending projections from the locus coerulueus^32^. Acute alcohol intoxication increases norepinephrine turnover and thereby decreases the tissue concentration of norepinephrine^72^, which may be sufficient to boost glymphatic function in the awake state^32^.

Interestingly, enlargement of perivascular spaces is a frequent finding in neuroimaging studies of chronic alcoholics^73^, consistent with the hippocampal atrophy noted above, and a general pattern of grey matter loss^74^. It is possible that structural changes in the perivascular space would adversely affect the fluid dynamics of the glymphatic system^75^.

The behavioral tests indicated that mice receiving 1.5 g/kg alcohol daily exhibited impaired learning when under the influence of alcohol, which persisted even 24 hours after withdrawal from alcohol, even though glymphatic function had by then normalized. Thus, the ongoing impairment in the behavior test may be due to CNS changes unrelated to glymphatic function. Alcohol is known to suppress activity of the cholinergic system of the basal forebrain, which has been linked to learning impairment in binge drinking experiments in adolescent mice^76^. Alternately, it is possible that restoration of glymphatic function may not yet have cleared all neurotoxic waste products that had accumulated during four weeks of continuous glymphatic suppression with daily alcohol.

In conclusion, the main finding of this study is that a low dose of ethanol, comparable to 2.6 daily drink equivalents (for a 70 kg person) per day, increases glymphatic function in mice, which is expected to facilitate clearance of metabolic waste and potentially toxic proteins from the interstitial fluid. This beneficial effect of light alcohol intake was linked to a decreased GFAP expression in astrocytes. We hypothesize that boosting of glymphatic function in combination with the reduction in GFAP expression might potentially contribute to the lowered risk for Alzheimer’s disease and non-Alzheimer’s dementia among individuals with habitually low but non-zero alcohol intake^8,9,17^.

## Methods

### Mice

All experiments were carried out according to relevant guidelines and regulations. All animal experiments were approved by the Animal Care and Use Committee at University of Rochester and carried out according to University of Rochester and Association for Assessment and Accreditation of Laboratory Animal Care (AAALAC) standards. Mice were housed in 12 hour light/dark cycle in groups of five with *ad libitum* access to standard chow pellets and water. Male C57Bl6 mice, 25–35 g, were randomized into groups receiving intraperitoneal (IP) injections of low, intermediate, and high doses of ethanol (0.5, 1.5 and 4 g/kg, respectively) at a concentration of 21% w/v in saline (0.9% sodium chloride) or saline as control. Ethanol was administered 15 min before acute experiments, or once a day for 30 days for chronic experiments. In chronic exposure experiments, the last ethanol treatment was administered either 15 minutes (chronic) or 24 hours (chronic, withdrawal) before the glymphatic tracer experiment.

### Immobility-defined sleep analysis

Mice were single housed and acclimatized to the cage for 72 hours in a 12 h:12 h light: dark room isolated from external stimuli. Sleep was recorded for 24 hours using Oxymax comprehensive Laboratory Animal Monitoring System (CLAMS, Columbus Instruments, Columbus, OH) which provides a scaffold of high resolution infra-red light beams equally spaced on the XY plane. Cages were placed within the scaffold/beam break system, and individual lids reduced the possibility of inter cage mouse activity noise to negligible. Any beam interuptions caused by movement (ambulatory counts) were recorded and analyzed with the help of linked Oxymax V5.35 software and associated algorithms (Columbus instruments, Columbus, OH). Sleep was defined by single epochs of 10 sec immobility with zero beam breaks^34^.

### Motor assessment

Mice were put on a Rota-Rod (Ugo Basile) that was set to accelerate from 5–40 rpm over a 15 minute trial period. A total of six trials were performed and the time to failure was averaged for each set of two trials, with at least 30 minute rest between trials. The experimenter was a female who had previously handled by the same experimenter several times per week, including on the same day of the Rota-Rod tests.

### Novel object test

The mice were placed in a 30 × 54 cm plastic bin and left to explore for ten minutes. Two plastic objects were added to the bin, and the mice then explored the objects for ten minutes. Each treatment group was divided into three sub-groups that were given different sets of objects. 60 minutes after the learning phase, one of the objects was replaced with an unfamiliar one. All trials were recorded by an overhead video camera, and the testing chamber was cleaned with 70% ethanol between each testing phase. The data for how long the mouse spent actively exploring each object was obtained using ANY-Maze software and verified by an experimenter visually inspecting the videos and the percentage of time spent examining the novel object was calculated as the percentage of the total time each mouse explored objects. Looking directly at the object and/or sniffing it was defined as “active exploration”; sitting next to, climbing on, or sleeping next to the object were not included in the measured time. Mice spending less than five seconds in total exploring objects were excluded from analysis^77^.

### Cisterna magna injections

The day before the experiment, the mice were anesthetized with anesthesia (100 mg, 10 mg, respectively, IP), and a 30 gauge needle connected to a closed-end PE10 tubing was implanted in the cisterna magna, and secured to the skull surface by superglue mixed with dental cement. All tracer experiments were performed in awake condition by injection of 5 μL Alexa647-conjugated bovine serum albumin (BSA-647) into the implanted cannula. This injection was performed using a Harvard Instruments syringe pump (Series 11 Elite), set at an infusion rate of 1 μL/min. Mice were restrained briefly before start of the injection, but injection of tracer and chase were performed in awake, freely moving mice. The total circulation time was 30 or 180 minutes from start of the BSA-647 injection. The mice were monitored continuously during the 30 or 180 min and were not allowed to sleep. Just 60 seconds before the end of the experiment the mice were rapidly anesthetized with inhalable isoflurane (2.5% isoflurane in 1 L oxygen/min) and decapitated. Brains were removed and immersion-fixed in 4% paraformaldehyde (PFA) in phosphate-buffered saline solution (PBS, Sigma) overnight at 4 °C, and cut the next day into 100 μm-thick coronal slices using a Leica VTS1000 vibratome. For the clearance experiments (180 min), the mice were perfused for two minutes with ice-cold PBS before the brain was removed. Brains from this experiment were separated into left and right hemisphere and one hemisphere was used for another analysis (see cytokine analysis) in order to reduce the number of mice used for these experiments. Since the fluorescence signal after 180 min is very low due to extensive clearance, these slices were counterstained with DAPI (Invitrogen).

### Immunohistochemistry

Under deep anesthesia, the mice were perfused transcardially with 4% PFA in PBS, and the brains were post-fixed overnight and cut coronally into 100 µm-thick slices on a vibratome. Slices were permeablilized with 0.1% Triton-X-100 in PBS, blocked with 7% normal donkey serum (Jackson Immunoresearch) in PBS with 0.03% Triton-X-100 and incubated with primary antibody overnight, followed by three washes in PBS and incubation with the fluorophore-linked secondary antibodies (Jackson Immunoresearch) for two hours. Stained slices were mounted with Fluoromount G (Thermofischer Scientific). Primary antibodies used were: rabbit anti-GFAP (AB5804, Millipore) and rabbit anti-AQP4 (50-173-540, Fisher Scientific), cell nuclei were identified using DAPI (Invitrogen).

### Imaging and quantification

The BSA-647 tracer in brain slices was imaged using a 0.63 × lens with 2X magnification on an MvX10 microscope (Olympus) using a Lumencore 1600 (Prior) light source with Metamorph Basic (Olympus) software and saved in 16 bit TIFF format. Images were quantified by ImageJ software version 1.47 (National Institutes of Health, imagej.nih.gov/ij/). The percent of area covered by tracer was determined using a pre-established threshold function. Immmunostainings were imaged with a 40×/1.3 magnification on a confocal microscope (IX81, Olympus). The mean immunofluorescence intensity was quantified using ImageJ. For quantification of AQP4 polarization, representative 50 μm segments centered around blood vessels were analyzed using the line-plot tool in ImageJ. For polarity calculation, the peak intensity of the vascular end-feet was divided by the average of the baseline.

### Cytokine assay

The mice were anesthetized with isoflurane and were transcardially perfused with ice-cold PBS for two minutes so that blood was cleared from the liver, and implicitly from brain. The brains were quickly removed and cut sagitally, by the midline. One hemisphere was snap-frozen and kept in the −80 °C freezer. The brains were homogenized by sonication on ice in a buffer containing 20 mM Tris pH 7.4, 150 mM NaCl, 0.5% Tween-20, and a 1:100 dilution of protease/phosphatase inhibitor (Sigma, P8340) in PBS and centrifuged at 4 °C at 13,000 *g* for 15 minutes. The cytokines were measured using the Multiplex Laser Bead platform by Eve Technologies (Alberta, Canada) or AssayGate (Maryland, USA). In order to fulfill our obligation to minimize the number of mice used for experiments, we combined the cytokine assay with a glymphatic assay for clearance. The mice had received a CM injection with Alexa-647 dye. In order to check that the Alexa-647 dye did not interfere with the fluorescent measurements, we sent test samples consisting of three samples of mouse brain without tracer and three samples of mouse brain with tracer. The results showed that the cytokine measurements did not differ between these two groups (p > 0.05 for all cytokines).

### Statistics

All statistical analyses were performed using Graph Pad Prism software version 7. Groups means were compared using one-way or two-way ANOVA followed by Dunnett’s or Tukey’s test, as appropriate. Values are expressed as mean ± SEM. P-values denoted as *p < 0.05, **p < 0.01, ***p < 0.001.

## Electronic supplementary material

Supplementary information
