# Supplementary material for: Beneficial effects of low alcohol exposure, but adverse effects of high alcohol intake on glymphatic function
Source: Sci Rep. 2018 Feb 2;8:2246. doi: 10.1038/s41598-018-20424-y (PMC5797082; doi:10.1038/s41598-018-20424-y)
Supplement: Supplementary file 1 — Supplementary information [file 41598_2018_20424_MOESM1_ESM.pdf]

Supplemental information to: Beneficial effects of low alcohol exposure, but adverse effects of high alcohol intake on glymphatic function

Iben Lundgaard[1], Wei Wang[1], Allison Eberhardt[1], Hanna Sophia Vinitsky[1], Benjamin Cameron Reeves[1], Sisi Peng[1,2], Nanhong Lou[1], Rashad Hussain[1], Maiken Nedergaard[1,3]

[1]Center for Translational Neuromedicine, Department of Neurosurgery, University of Rochester, NY 14642, USA

[2]Department of Radiology, Tongji Hospital, Tongji Medical College, Huazhong University of Science and Technology, Wuhan 430030, China.

[3]Center for Basic and Translational Neuroscience, University of Copenhagen, 2200 Copenhagen, Denmark

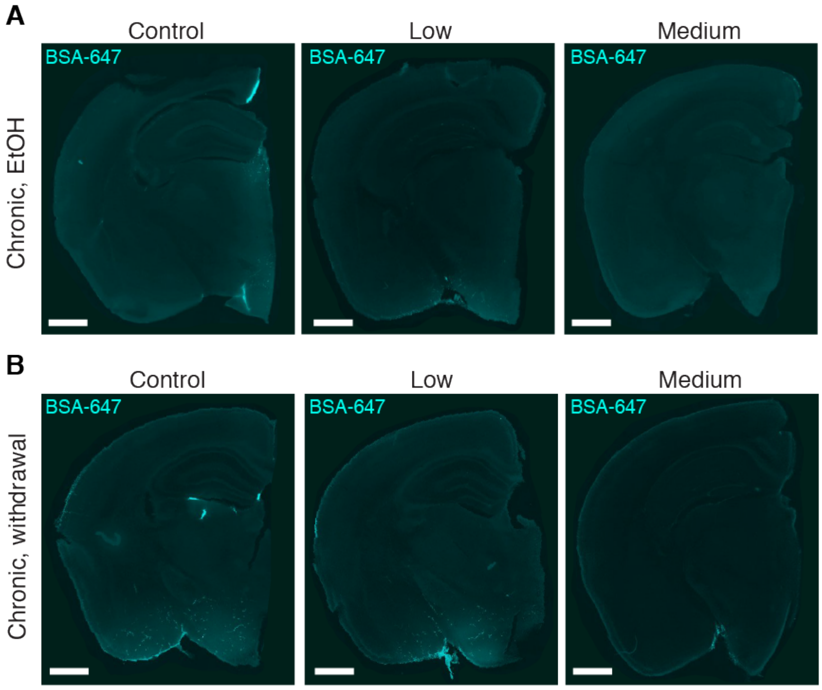

Supplemental Figure 1

Supplemental Figure 1. Tracer clearance is improved in alcohol-treated mice

Images from Figure 3 shown without DAPI. CSF tracer in coronal slices of left brain hemisphere at 180 min after injection in saline (CTR), low and medium dose of chronic alcohol with the last dose of alcohol given (A) immediately before and (**B**) given 24 hours before CM tracer injection with bovine serum albumin-Alexa647 (BSA-647).

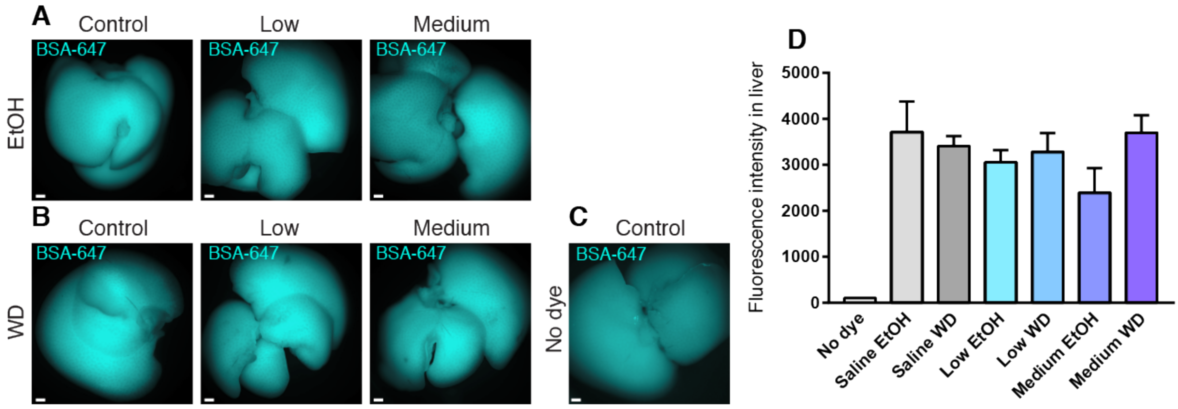

Supplemental Figure 2

Supplemental figure 2. Efflux of CSF tracer to liver is not affected by alcohol

A) Images of bovine serum albumin–Alexa647 (BSA-647) in livers of mice that underwent chronic alcohol treatment ending on the same day as alcohol (EtOH), (**B**) 24 hours later (withdrawal, WD) or (**C**) mice that were not injected with BSA-647 in the CM and (**D**) quantification thereof. One-way ANOVA, ns, not significant, between all six BSA-647-injected groups. Quantification of mice with no BSA-647 shown for comparison. Scale Bars, 1 mm. Bar graphs and plots represent mean and standard error of the mean (SEM) of 3 mice per group for mice not injected with BSA-647, 5 mice for saline and 9-11 mice for alcohol-treated mice.
